# Parallel activation of helicopter and ground transportation after dispatcher identification of suspected anterior large vessel occlusion stroke in rural areas: a proof-of-concept case with modeling from the LESTOR trial

**DOI:** 10.1186/s13049-024-01233-x

**Published:** 2024-07-06

**Authors:** Max Henningsen, Matthias L. Herrmann, Simone Meier, Ulrike Bergmann, Hans-Jörg Busch, Christian A. Taschner, Jochen Brich

**Affiliations:** 1 Department of Neurology and Neuroscience, Faculty of Medicine and Medical Center, University of Freiburg, Breisacher Str. 64, Freiburg, Germany; 2 Department of Emergency Medicine, Faculty of Medicine and Medical Center, University of Freiburg, Freiburg, Germany; 3 Department of Neuroradiology, Faculty of Medicine and Medical Center, University of Freiburg, Freiburg, Germany

## Abstract

**Background:**

When stroke patients with suspected anterior large vessel occlusion (aLVO) happen to live in rural areas, two main options exist for prehospital transport: (i) the drip-and-ship (DnS) strategy, which ensures rapid access to intravenous thrombolysis (IVT) at the nearest primary stroke center but requires time-consuming interhospital transfer for endovascular thrombectomy (EVT) because the latter is only available at comprehensive stroke centers (CSC); and (ii) the mothership (MS) strategy, which entails direct transport to a CSC and allows for faster access to EVT but carries the risk of IVT being delayed or even the time window being missed completely. The use of a helicopter might shorten the transport time to the CSC in rural areas. However, if the aLVO stroke is only recognized by the emergency service on site, the helicopter must be requested in addition, which extends the prehospital time and partially negates the time advantage. We hypothesized that parallel activation of ground and helicopter transportation in case of aLVO suspicion by the dispatcher (aLVO-guided dispatch strategy) could shorten the prehospital time in rural areas and enable faster treatment with IVT and EVT.

**Methods:**

As a proof-of-concept, we report a case from the LESTOR trial where the dispatcher suspected an aLVO stroke during the emergency call and dispatched EMS and HEMS in parallel. Based on this case, we compare the provided aLVO-guided dispatch strategy to the DnS and MS strategies regarding the times to IVT and EVT using a highly realistic modeling approach.

**Results:**

With the aLVO-guided dispatch strategy, the patient received IVT and EVT faster than with the DnS or MS strategies. IVT was administered 6 min faster than in the DnS strategy and 22 min faster than in the MS strategy, and EVT was started 47 min earlier than in the DnS strategy and 22 min earlier than in the MS strategy.

**Conclusion:**

In rural areas, parallel activation of ground and helicopter emergency services following dispatcher identification of stroke patients with suspected aLVO could provide rapid access to both IVT and EVT, thereby overcoming the limitations of the DnS and MS strategies.

**Supplementary Information:**

The online version contains supplementary material available at 10.1186/s13049-024-01233-x.

## Background

In large vessel ischemic stroke, 1.9 million neurons are lost every minute that the patient remains untreated [1]. Therefore, it is highly important to initiate recanalization therapy as soon as possible [2, 3]. If feasible, stroke patients with anterior large vessel occlusion (aLVO) should be treated with both intravenous thrombolysis (IVT) and endovascular thrombectomy (EVT) [4, 5]. As in many other countries, in Germany, IVT can be carried out at any primary stroke center (PSC), whereas EVT is available only at comprehensive stroke centers (CSC).

To date, the vast majority of emergency medical services (EMS) apply two different transport strategies for aLVO stroke patients: the drip-and-ship (DnS) strategy and the mothership (MS) strategy. In rural areas, neither of them can ensure rapid access to both IVT and EVT. The DnS strategy with transport to the nearest primary stroke center allows for fast administration of IVT but requires time-consuming interhospital transfer to the CSC for EVT [6]. In contrast, the MS strategy with direct transport to the distant CSC and bypassing of the local PSC entails fast access to EVT [7], but the administration of IVT might be delayed or even impossible because the time window has been exceeded. In rural areas where the CSC is located far away, helicopters are often used in Germany for the fastest transport to the CSC. However, the subsequent request for helicopter emergency medical service (HEMS) by ground EMS, including the dispatch and arrival of the helicopter and the handover of the patient from EMS to HEMS, prolongs the on-scene time. As a result, IVT at the CSC is still at least partially delayed compared to the DnS strategy.

Parallel activation of both EMS and HEMS at the start of the emergency response in the case of aLVO suspicion by the dispatcher (aLVO-guided dispatch strategy) could shorten the prehospital time by preserving the usual ʻtime on sceneʼ without requiring extra time for the subsequent request of a helicopter [8, 9]. Given that air-based transport to the CSC is rarely longer than ground-based transport to the next PSC in our area, we hypothesized that this strategy could enable faster time-to-treatment in CSCs for both EVT and IVT.

## Methods

### Proof-of-concept

We present a proof-of-concept case of a stroke patient living in a rural area who was correctly identified by the dispatcher as having an aLVO stroke during the emergency call and therefore received parallel activation of EMS and HEMS. The reported case is the first patient who was provided with the aLVO-guided dispatch strategy in rural areas as part of the LESTOR trial (German Clinical Trails Register ID: DRKS00022152). This trial aims to investigate the feasibility of identifying suspected aLVO stroke patients during emergency calls and the impact of the resulting dispatch optimization on the clinical outcome [10]. In the aLVO-guided dispatch strategy, regular stroke detection with the FAST (Face, Arm, Speech, Time) test is followed by an aLVO-query which has been newly developed specifically for dispatchers [10]. This aLVO-query consists of a step-by-step examination performed by the emergency caller under the guidance of the dispatcher and aims to detect a combination of an arm paresis and a correspondent cortical sign (i.e. gaze deviation, neglect and/or aphasia).

### Case-based model

**Fig. 1:**
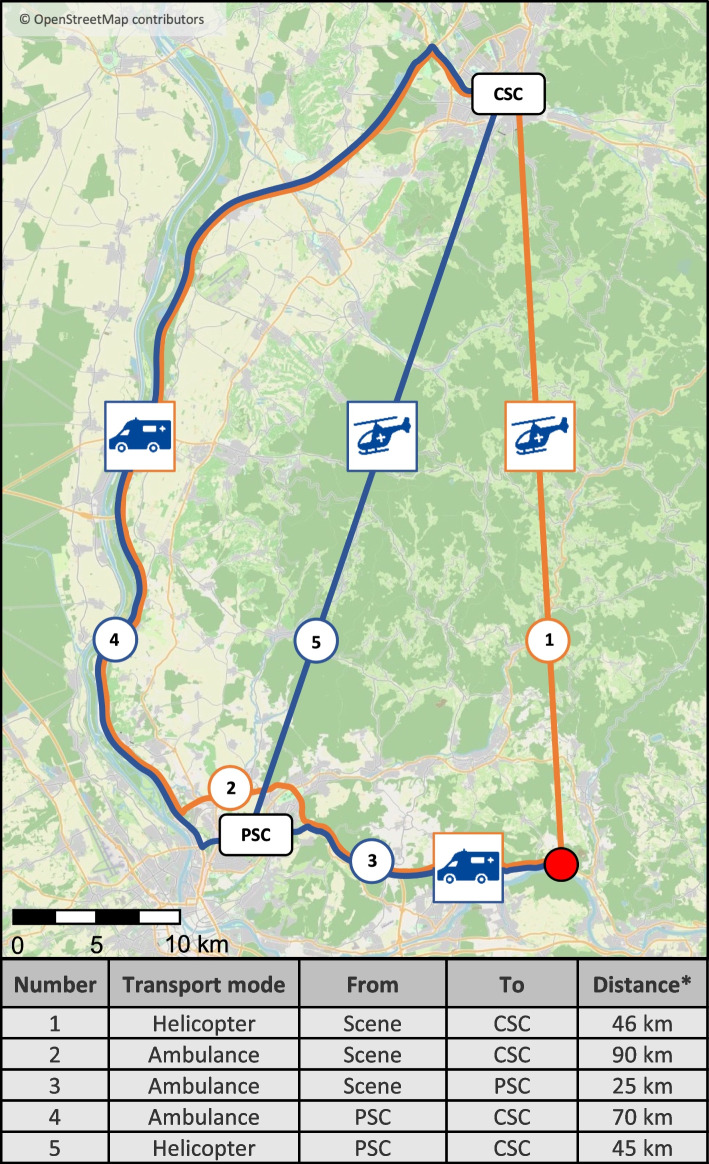
Emergency medical service transport options and distances. Red dot: emergency scene. Abbreviations: CSC, comprehensive stroke center; PSC, primary stroke center. *Air-line distance in the case of helicopter transport. This map was generated using OpenStreetMap, which is available under the Open Database License (© OpenStreetMap contributors)

We compare the ʻaLVO-guided dispatch strategyʼ to the common DnS and MS strategies in terms of prehospital time intervals and time-to-treatment in a realistic case-based model. In the DnS and MS strategies, transport can be carried out with helicopter (ʻairʼ) or ambulance (ʻgroundʼ). For a detailed presentation of the transport options and distances, see Fig. 1. We modeled prehospital timelines by combining original EMS data from the case, highly realistic EMS routing data for alternative routes, and default times for prehospital and in-hospital stroke care. Ambulance travel times with lights and siren use and helicopter interhospital flight times were provided by rescuetrack, a routing service that is also used by local EMS and HEMS (rescuetrack GmbH, Reutlingen, Germany, https://rescuetrack.com). The specific mapbutler™ routing service that was used for this analysis extracts information about road connectivity (topology) from static road network data (OpenStreetMap data in this case). The expected travel speeds are based on a combination of road-attributed data and Floating Car Data, as provided by the Automatic Vehicle Location Service component of their product, which processes telemetry data from emergency service vehicles that use their service. The current analysis used a routing product with static weights independent of parameters such as time of day and congestion level. This provided a comparable, averaged baseline for the reachability analysis and other planning applications. Similarly, the helicopter routing product uses a travel speed profile developed in collaboration with HEMS providers and verified using real-world telemetry data. Default times were set according to national target times (prehospital times: on-scene time = 30 min; in-hospital times: door-to-needle at PSC and CSC = 30 min, needle-to-door at PSC = 30 min, door-to-groin puncture at CSC = 60 min in the case of DnS, needle-to-groin puncture at CSC = 60 min in the case of MS) and empirical values generated from our stroke network (subsequent request of helicopter by EMS = 20 min after arrival at scene, patient handover time between EMS and HEMS = 15 min). A detailed presentation of the modeling scheme can be found in Additional file 1 (Figure S1 and Table S1).

## Results

### Emergency situation

A 56-year-old male patient developed acute left-sided hemiparesis and neglect. The patient had no stroke in his previous medical history and suffered from diabetes and hypertension. Symptom onset occurred at 5.15 pm. The emergency scene was located in a village with 2500 inhabitants in the southern Black Forest region. The closest EMS base was located 8 km away by ambulance, while the next HEMS base was 26 km away by helicopter. The driving distance from the emergency scene to the closest PSC was 26 km, while the CSC was 90 km away. The air-line distance to the CSC was 46 km.

### Prehospital care

The emergency call was received 24 min after symptom onset (5:39 pm) and the dispatcher's standard emergency assessment was completed within 3 min (5:42 pm). The dispatcher suspected a stroke and dispatched EMS, which immediately departed ambulance base 1 (for EMS and HEMS base locations, see Figure S2 in Additional file 1). The dispatcher continued the emergency call with the aLVO-query, revealing a combination of left-arm paresis and gaze deviation to the right, and hence indicating a possible aLVO stroke. HEMS was additionally dispatched (5:44 pm) and departed immediately from helicopter base 2. EMS personnel arrived at scene 10 min after dispatch (5:52 pm), and the helicopter landed close to scene 4 min later (5:56 pm). On-scene EMS personnel confirmed the suspicion of aLVO, and helicopter transport to the next CSC was initiated (take-off from scene at 6:22 pm). The total flight time to the closest CSC, including take-off and landing, was 14 min.

### Diagnostic assessment, therapeutic intervention and outcome

The patient arrived at the CSC at 6:36 pm (57 min after emergency call was received and 44 min after EMS arrival at emergency scene). The National Institutes of Health Stroke Scale (NIHSS) score was 11 (gaze deviation to the right, left-sided hemianopsia, left-sided hemiparesis, dysarthria, left-sided neglect). Noncontrast computed tomography (CT) imaging ruled out intracranial hemorrhage and showed only minimal early signs of infarction in the right middle cerebral artery territory (Alberta Stroke Program Early CT Score [ASPECTS] of 9), so IVT was started. CT angiography revealed proximal occlusion of the dominant M2 segment of the right middle cerebral artery, and the patient was immediately transferred to the angiography suite. EVT achieved successful recanalization (Thrombolysis in Cerebral Infarction [TICI] grade: 2b). The patient was discharged to his home. NIHSS and Modified Rankin Score (mRS) were 0 at discharge.

### Comparison to alternative transport strategies

**Fig. 2:**
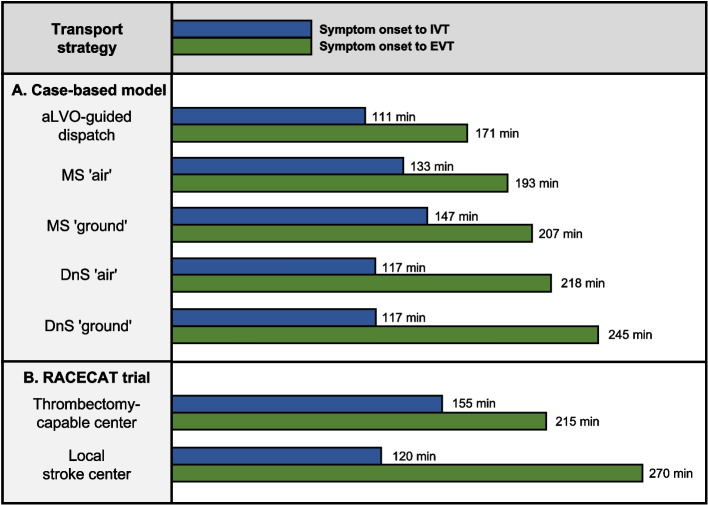
Time from symptom onset to recanalization therapy (IVT and EVT) in A. case-based modeled transport strategies and B. comparison to median times of the RACECAT trial [7]. RACECAT trial times are presented as the median value. Note that in the RACECAT trial, primary and interhospital transport was almost exclusively ground-based (ʻThrombectomy-capable centerʼ therefore best corresponds to ʻMS groundʼ and ʻLocal stroke centerʼ best corresponds to ʻDnS groundʼ). For a detailed description of the modeling scheme, see Figure S1 and Table S1 in Additional file 1. Abbreviations: aLVO, anterior large vessel occlusion; DnS, drip-and-ship; MS, mothership; IVT, intravenous thrombolysis; EVT, endovascular thrombectomy

In the aLVO-guided dispatch strategy, EMS and HEMS arrived at the emergency scene almost simultaneously. In detail, the helicopter arrived at emergency scene 17 min after emergency call receipt, and only 4 min after EMS arrival at scene. In comparison, in the MS ʹairʹ strategy, the helicopter would have been requested by ground EMS 20 min after their arrival at scene and thus would have first arrived at scene 50 min after the initial emergency call receipt. This would have significantly increased the total on-scene time in the MS ʹairʹ strategy (52 min). In the aLVO-guided dispatch strategy, the helicopter arrived at CSC faster than the ambulance would have arrived at PSC in the DnS strategy. Table 1 shows a detailed comparison between the patient's time flow until arrival at the CSC and the modeled alternative transport strategies. The use of a helicopter would generally shorten the transport time to CSC in the context of both the MS and DnS strategies (-14 min in the MS strategy, and -27 min in the DnS strategy). Nevertheless, the aLVO-guided dispatch strategy would enable even faster access to both IVT and EVT than the air-based DnS and MS strategies (see Fig. 2). More specifically, IVT started 6 min earlier when compared to what was previously the fastest strategy for IVT (DnS), while EVT also started earlier (22 min) in comparison to the start-time of the previously fastest strategy for EVT, MS ʻairʼ.

**Table 1:** Comparison of patient transport time to CSC (aLVO-guided dispatch) with realistically modeled alternative transport strategies

Transport strategy	aLVO-guided dispatch	MS ʹairʹ	MS ʹgroundʹ	DnS ʹairʹ	DnS ʹgroundʹ
**Mode of transport**					
To PSC	-	-	-	ambulance	ambulance
To CSC	helicopter	helicopter	ambulance	helicopter	ambulance
**Time from symptom onset to**					
Emergency call receipt	24	24	24	24	24
EMS dispatch	27	27	27	27	27
HEMS dispatch	29	-	-	-	-
Ambulance arrival at scene	37	37	37	37	37
Subsequent request of helicopter	-	57	-	-	-
Helicopter arrival at scene	41	74	-	-	-
Start of ambulance transport	-	-	67	67	67
Start of helicopter transport	67	89	-	-	-
Arrival at PSC	-	-	-	87	87
Ambulance departure from PSC	-	-	-	-	147
Helicopter departure from PSC	-	-	-	147	-
Arrival at CSC	**81**	**103**	**117**	**158**	**185**

All times are shown in minutes. In the MS ʻairʼ strategy, the subsequent request of helicopter is made 20 min after EMS arrival, and the patient handover time between EMS and HEMS was set to 15 min (based on empirical data from our stroke network. *Abbreviations: aLVO* anterior large vessel occlusion, *MS* mothership, *DnS* drip-and-ship, *PSC* primary stroke center, *CSC* comprehensive stroke center, *EMS* emergency medical service, *HEMS* helicopter emergency medical service

## Discussion

Here, we present real-world data of a prehospital transport strategy adjusted for suspected aLVO stroke patients in rural areas (ʻaLVO-dispatch strategyʼ) that results in faster access not only to EVT but also to IVT compared to highly realistic modeled alternative transport strategies.

While the time from stroke onset to emergency call is difficult to influence [11, 12], the time that lapses from emergency call to recanalization therapy (IVT and EVT) can be positively affected by optimizing the prehospital workflow. A number of factors significantly impact the prehospital time: (i) the initial dispatch of rescue means based on the emergency call, (ii) the stroke patient transport strategy based on preclinical assessment at the scene, and (iii) the mode of transport to the target hospital based on the distance between the scene and the hospital and the availability of specific emergency transport. The two common allocation strategies for aLVO stroke patients both have significant advantages and disadvantages: the MS strategy is known for fast arrival at the CSC and thus fast access to EVT, but the delayed or even aborted initiation of IVT reduces its benefits. On the other hand, the DnS strategy allows for faster access to IVT, but is associated with delayed EVT. In fact, the recent RACECAT trial comparing MS and DS strategies in suspected LVO stroke patients in non-urban areas in Catalonia (Spain) showed no significant difference in 90-day disability [7]. It is noteworthy that in this comparison, despite long transport distances, transport to CSC was almost exclusively carried out ground-based, resulting in travel times to the next CSC > 60 min in more than half of the study population. While the assumed treatment times for MS ʻgroundʼ and DnS ʻgroundʼ in our example were remarkably consistent with those observed in the RACECAT trial (see Fig. 2), the use of the secondary requested helicopter as part of the MS ʻairʼ strategy would already shorten the prehospital time in the case of transport to a CSC by 14 min compared to ground-based transport, enabling IVT after 133 min and EVT after 193 min instead of 147 min and 207 min, respectively. Nevertheless, the time to IVT would still be delayed by 16 min with the MS ʻairʼ strategy compared to the DnS strategy.

To further optimize the prehospital workflow, parallel dispatch of ground EMS and HEMS in the case of aLVO stroke suspicion by the dispatcher (aLVO-guided dispatch) could save time on site as the helicopter is immediately available for transport without the need of a subsequent request. Emergency rescue data from a German air-rescue study showed that parallel activation of EMS and HEMS shortens the on-scene time: while the median on-scene time was 53 min in the case of secondary request of helicopter, the median on-scene time with parallel activation only amounted to 28 min [13]. Since only 10% of cases in this study accounted to “neurological emergencies”, there is a lack of real-world data investigating the parallel activation of EMS and HEMS in patients with aLVO stroke.

Based on our case, we compared different prehospital strategies with the aLVO-guided dispatch strategy. The calculated time differences between the different strategies could be clinically highly relevant, as a recent post hoc analysis of the RACECAT trial demonstrated that time differences very similar to those observed in our comparison led to significant differences in clinical outcome [14]. Overall, it can be assumed that the time benefit of the aLVO-guided dispatch strategy would further increase with greater transport distance since not only the driving distance to the CSC but also the flight distance from the helicopter base to the emergency scene increases. The parallel activation of HEMS warrants that the helicopter approach to the scene starts at the earliest possible time point and does not extend the on-scene time, as in the case of secondary helicopter request in the MS ʻairʼ strategy. Despite long transport distances, the aLVO-guided dispatch strategy would ensure timely administration of IVT. This finding appears particularly important in light of a recent meta-analysis showing that the benefit of IVT in combination with EVT versus EVT alone is linearly time-dependent, with a statistically significant benefit demonstrated only when the time from symptom onset to IVT administration was less than 140 min [15]. These results once again emphasize the need to initiate IVT as early as possible also in aLVO stroke patients to obtain the greatest possible clinical benefit, which is best enabled by the aLVO-guided dispatch strategy.

Moreover, the aLVO-guided dispatch strategy could be beneficial for patients with intracerebral hemorrhage (ICH), who, particularly in the case of cortical lobar hemorrhage, can present with the same symptoms as aLVO stroke patients and therefore cannot be distinguished by the dispatcher. These patients benefit from immediate access to neurosurgical care [16], which in our region is only available at CSCs. The aLVO-guided dispatch strategy could overcome the reported negative effects of long-distance ground transport to the CSC in patients with ICH from the RACECAT trial [17], not only because transport times are significantly shorter with helicopter but also because the constant presence of physicians in the helicopter allows improved medical support during patient transfer compared to the limited medical support provided by EMS.

This study has limitations. Since it is not possible to observe the timelines of different transport strategies on a single case basis, we applied modeling as a means to compare prehospital transport strategies. Modeling generally involves assumptions rather than real-time data. We were able to calculate highly realistic prehospital transport times for alternative transport strategies based on real-world emergency ambulance travel times and helicopter flight times. The default on-scene time of 30 min represents the median on-scene time in stroke rescue missions in our federal state (and is also exactly in line with the on-scene time of the present case) [18]. The default door-in-door-out time of 60 min for the DnS strategy represents an optimally functioning system. The real-world door-in-door-out times in our region [19], as well as in other countries [20, 21], considerably exceed this benchmark, so our modeled data might underestimate the time advantage of the aLVO-guided dispatch strategy relative to the DnS strategies. It should be noted that cross-border transport is not considered in our study as it is not regularly performed due to organizational matters and insurance regulations. Despite this, in our case, ambulance transport to the nearest foreign CSC (ambulance travel time from emergency scene: 24 min) would not have a time advantage over the aLVO-guided dispatch strategy.

There are also limitations associated with the aLVO-guided dispatch strategy itself. The aLVO-guided dispatch strategy requires the detection of suspected aLVO stroke patients in emergency calls. Detection of suspected aLVO stroke over the phone might be challenging, and the limited reliability of stroke detection by dispatchers could influence the success of this strategy [22]. The development of an aLVO-query specifically tailored for dispatchers is an important cornerstone of the LESTOR study [10]. The aLVO-query aims to recognize a combination of arm paresis and correspondent cortical sign as cortical symptoms have proved to be a reliable indicator for LVO [23]. A poor specificity of the aLVO-query could potentially lead to inefficient use or even overload of HEMS carrying the risk of disadvantaging other time-critical emergencies. Nevertheless, aLVO stroke and its mimics are a rare occasion, particularly in sparsely populated rural areas.

The availability and costs of HEMS could also pose a challenge to the aLVO-guided dispatch strategy. In Germany, helicopters are evenly distributed throughout the country [24], so the rare unavailability of helicopters is most often due to unfavorable weather conditions or limited night-flying capability. In our region, there are helicopters that have full night-flying capability and are able to land at unmarked landing sites even at night. The deployment of HEMS is checked regularly for transport distances of more than 40–50 km or in areas that are difficult to access on roads. Using helicopter transport for shorter distances might overstretch the HEMS system. In addition, since the number of available helicopters may be limited in other countries, prolonged helicopter approach times must be considered when selecting the fastest means of transport. A recent simulation study in Finland showed that helicopter transport of stroke patients was faster only when the estimated ground transport time exceeded 40 min, provided that the helicopter was dispatched in parallel [9]. Moreover, helicopter deployment is more expensive than ground transportation. Therefore, the LESTOR trial is accompanied by a cost-effectiveness analysis, which offsets the costs of helicopter missions against the savings resulting from the faster initiation of therapy.

## Conclusion

In conclusion, our proof-of-concept case demonstrates that the aLVO-guided dispatch strategy with parallel activation of EMS and HEMS provides rapid access to IVT and EVT in rural areas, potentially enabling better care of aLVO stroke patients than the DnS or MS strategies.

### Supplementary Information

Supplementary Material 1.

## Data Availability

All data generated or analyzed during this study are included in this published article and its additional file.

## Abbreviations

aLVO: Anterior large vessel occlusion
CSC: Comprehensive stroke center
CT: Computed tomography
DnS: Drip-and-ship
EMS: Emergency medical service
EVT: Endovascular thrombectomy
HEMS: Helicopter emergency medical services
ICH: Intracerebral hemorrhage
IVT: Intravenous thrombolysis
MS: Mothership
PSC: Primary stroke center
