# Supplementary material for: Parallel activation of helicopter and ground transportation after dispatcher identification of suspected anterior large vessel occlusion stroke in rural areas: a proof-of-concept case with modeling from the LESTOR trial
Source: Scand J Trauma Resusc Emerg Med. 2024 Jul 6;32:62. doi: 10.1186/s13049-024-01233-x (PMC11227698; doi:10.1186/s13049-024-01233-x)
Supplement: Supplementary file 1 — Supplementary Material 1. [file 13049_2024_1233_MOESM1_ESM.docx]

**Additional File**

**Parallel activation of helicopter and ground transportation after dispatcher identification of suspected anterior large vessel occlusion stroke in rural areas:**

**A proof-of-concept case with modeling from the LESTOR trial**

*Max Henningsen ^1^, Matthias L Herrmann ^1^, Simone Meier ^1^, Ulrike Bergmann ^1^,*

*Hans-Jörg Busch ^2^, Christian A Taschner ^3^, Jochen Brich ^1^*

^1^ Department of Neurology and Neuroscience, Faculty of Medicine and Medical Center, University of Freiburg, Freiburg, Germany

^2^ Department of Emergency Medicine, Faculty of Medicine and Medical Center, University of Freiburg, Freiburg, Germany

^3^ Department of Neuroradiology, Faculty of Medicine and Medical Center, University of Freiburg, Freiburg, Germany

**Corresponding author:**

Dr Max Henningsen, Department of Neurology and Neuroscience, Faculty of Medicine and Medical Center, University of Freiburg, Breisacher Str. 64, 79106 Freiburg, Germany; max.henningsen@uniklinik-freiburg.de

**Figure S1 Prehospital and in-hospital workflows of the aLVO-guided dispatch strategy and common alternative transport strategies.**


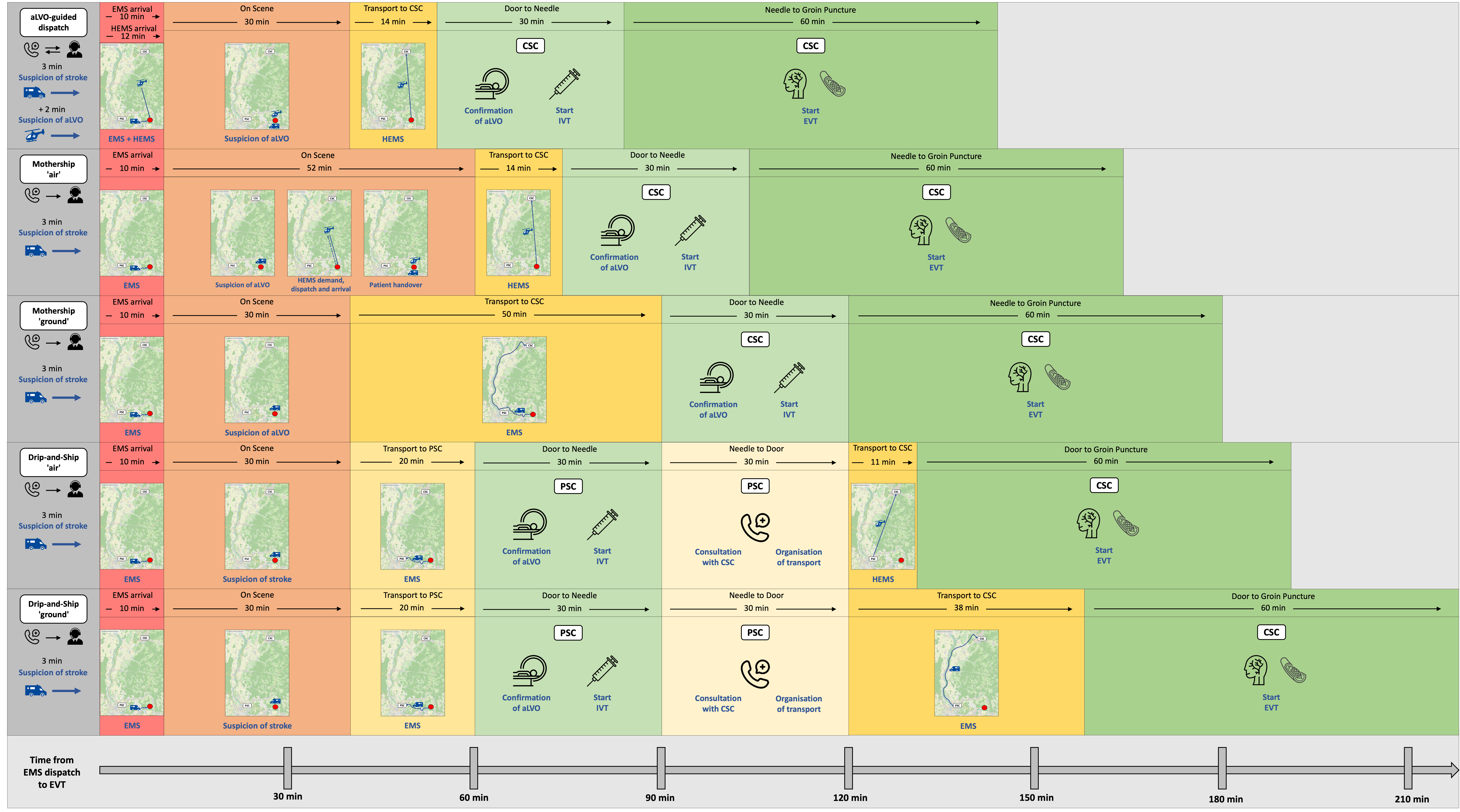


aLVO, anterior large vessel occlusion; EMS, emergency medical service; HEMS, helicopter emergency medical service; PSC, primary stroke center; CSC, comprehensive stroke center; IVT, intravenous thrombolysis; EVT, endovascular thrombectomy. Maps were generated using OpenStreetMap, which is available under the Open Database Licence (© OpenStreetMap contributors).

**Figure S2 Location of emergency medical service ambulance and helicopter bases and distances to the emergency scene.**

**
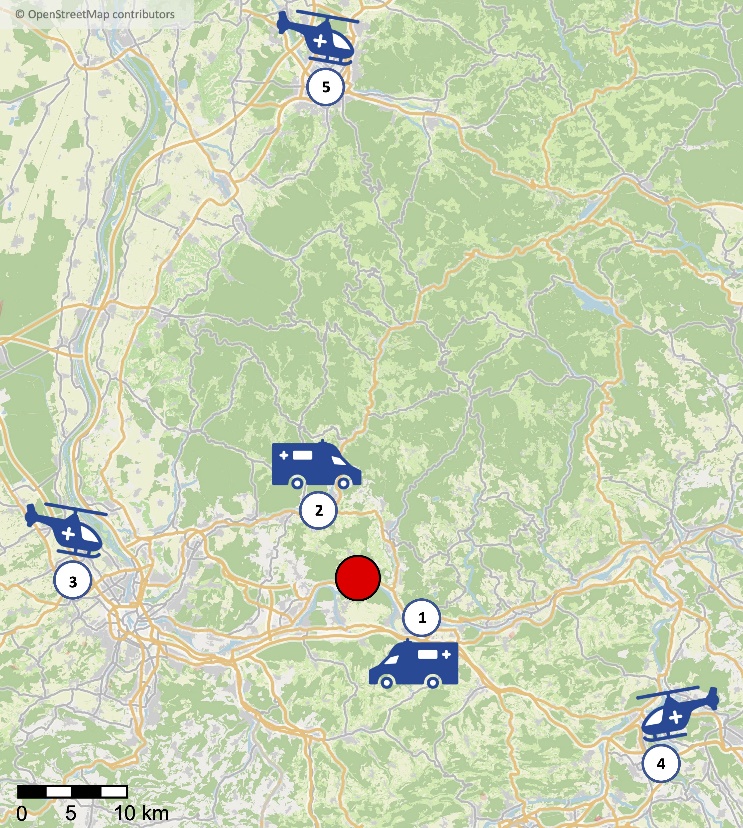
**

| **Number** | **Transport mode** | **From** | **To** | **Distance*** |
| --- | --- | --- | --- | --- |
| 1 | Ambulance | Ambulance base 1 | Emergency scene | 8 km |
| 2 | Ambulance | Ambulance base 2 | Emergency scene | 9 km |
| 3 | Helicopter ^#^ | Helicopter base 1 | Emergency scene | 26 km |
| 4 | Helicopter ^#^ | Helicopter base 2 | Emergency scene | 32 km |
| 5 | Helicopter | Helicopter base 3 | Emergency scene | 47 km |

*Air-line distance in case of helicopter transport. CSC, comprehensive stroke center; PSC, primary stroke center. This map was generated using OpenStreetMap, which is available under the Open Database Licence (© OpenStreetMap contributors).

^#^ These helicopters are part of the Swiss helicopter emergency service system but can be activated by the German disptachers.

**Table S1 Composition and reference of time intervals in the case-based model.**

Green boxes indicate original time intervals originating from the EMS documentation of the real case. Blue boxes indicate highly realistic time intervals determined from real-world emergency routing data. Yellow boxes indicate default time intervals derived from national target times or empirical values from our stroke network.

| **Transport strategy** | **aLVO-guided dispatch** | **MS**  **ʹairʹ** | **MS**  **ʹgroundʹ** | **DnS**  **ʹairʹ** | **DnS**  **ʹgroundʹ** |
| --- | --- | --- | --- | --- | --- |
| Symptom onset  to EMC receipt | 24 | 24 | 24 | 24 | 24 |
| EMC receipt  to EMS arrival at scene | 13 | 13 | 13 | 13 | 13 |
| EMS arrival at scene to  HEMS departure from scene  (on-scene time) | 30 | 52 ***** | - | - | - |
| EMS arrival at scene to  EMS departure from scene  (on-scene time) | - | - | 30 ^°^ | 30 ^°^ | 30 ^°^ |
| HEMS depature from scene  to arrival at CSC | 14 | 14 | - | - | - |
| EMS depature from scene  to arrival at CSC | - | - | 50 | - | - |
| EMS depature scene  to arrival at PSC | - | - | - | 20 | 20 |
| Door-to-needle at PSC | - | - | - | 30 | 30 |
| Needle-to-door at PSC | - | - | - | 30 | 30 |
| HEMS departure from PSC  to arrival at CSC | - | - | - | 11 | - |
| EMS departure from PSC  to arrival at CSC | - | - | - | - | 38 |
| Door-to-needle at CSC | 30 | 30 | 30 | - | - |
| Door-to-groin puncture at CSC | 90 | 90 | 90 | 60 ^#^ | 60 ^#^ |
| **Symptom onset to IVT** | **111** | **133** | **147** | **117** | **117** |
| **Symptom onset to EVT** | **171** | **193** | **207** | **218** | **245** |

aLVO, anterior large vessel occlusion; MS, mothership; DnS, drip-and-ship; EMC, emergency medical call; EMS, emergency medical service; HEMS, helicopter emergency medical service; PSC, primary stroke center; CSC, comprehensive stroke center; IVT, intravenous thrombolysis; EVT, endovascular thrombectomy.

* In the MS ʻairʼ strategy, on-scene time comprised the time from EMS arrival at the scene to the secondary demand for a helicopter (20 min, empirical value from our stroke network), the helicopter arrival time (17 min, originating from the real case), and the patient handover time between EMS and HEMS (15 min, empirical data from our stroke network). Non-stroke-specific analysis of emergency operations with secondary demand for helicopter in Germany showed comparable on-scene times (Gries A, Lenz W, Stahl P, Spiess R, Luiz T. Präklinische Versorgungszeiten bei Einsätzen der Luftrettung: Einfluss der Dispositionsstrategie der Rettungsleitstelle. Anaesthesist. 2014;63(7):555-562).

° The default time of 30 min that was used for EMS arrival at the scene until departure (= on-scene time) was exactly in line with the measured on-scene time of the reported case. German EMS works according to a national standard procedure, that includes an extensive anamnesis and clinical evaluation of stroke symptoms as well as oxygen, blood pressure and blood sugar diagnostics and, if necessary, therapies.

# In case of DnS strategy, door-to-groin puncture time at CSC is only 60 min, since cerebral imaging and, if applicable, initiation of thrombolysis were already carried out at PSC.
